# BORIS, a paralogue of the transcription factor, CTCF, is aberrantly expressed in breast tumours

**DOI:** 10.1038/sj.bjc.6604181

**Published:** 2008-01-15

**Authors:** V D'Arcy, N Pore, F Docquier, Z K Abdullaev, I Chernukhin, G-X Kita, S Rai, M Smart, D Farrar, S Pack, V Lobanenkov, E Klenova

**Affiliations:** 1 Department of Biological Sciences, University of Essex, Wivenhoe Park, Colchester, Essex CO4 3SQ, UK; 2 Molecular Pathology Section, Laboratory of Immunopathology, National Institute of Allergy and Infectious Diseases, National Institutes of Health, (LIP/NIAID/NIH), Twinbrook Building, Room 1329, MSC-8152, 5640 Fisher Lane, Rockville, MD 20852, USA

**Keywords:** BORIS, CTCF, breast cancer, progesterone receptor, oestrogen receptor

## Abstract

BORIS (for brother of the regulator of imprinted sites), a paralogue of the transcription factor, CTCF, is a novel member of the cancer-testis antigen family. The aims of the present study were as follows: (1) to investigate BORIS expression in breast cells and tumours using immunohistochemical staining, western and real-time RT–PCR analyses and (2) assess potential correlation between BORIS levels in tumours with clinical/pathological parameters. BORIS was detected in all 18 inspected breast cell lines, but not in a primary normal breast cell culture. In 70.7% (41 of 58 cases) BORIS was observed in breast tumours. High levels of BORIS correlated with high levels of progesterone receptor (PR) and oestrogen receptor (ER). The link between BORIS and PR/ER was further confirmed by the ability of BORIS to activate the promoters of the *PR* and *ER* genes in the reporter assays. Detection of BORIS in a high proportion of breast cancer patients implies potential practical applications of BORIS as a molecular biomarker of breast cancer. This may be important for diagnosis of the condition and for the therapeutic use of BORIS. The ability of BORIS to activate promoters of the *RP* and *ER* genes points towards possible involvement of BORIS in the establishment, progression and maintenance of breast tumours.

BORIS (for brother of the regulator of imprinted sites) is a paralogue of the 11 zinc-finger transcription factor, CTCF. The zinc-finger domain of BORIS protein has striking homology to the CTCF zinc-finger domain; however, the N- and C-terminal domains of BORIS are different from these domains of CTCF (Klenova *et al*, 2002; Loukinov *et al*, 2002). BORIS is normally expressed only in spermatocytes in the testis; however, it is aberrantly expressed in various tumours and cancer cell lines (Klenova *et al*, 2002; Loukinov *et al*, 2002; Hoffmann *et al*, 2006; Risinger *et al*, 2007). Interestingly, BORIS was found in the leukocyte fraction of patients with breast cancer (D'Arcy *et al*, 2006).

BORIS can be classified as a protein belonging to the cancer testis antigen (CTA) family (Scanlan *et al*, 2004). The CTA gene products exhibit highly tissue-restricted expression and are immunogenic in cancer patients (Scanlan *et al*, 2002, 2004). The CTAs have been grouped into 44 families and include up to 89 CTA genes or isoforms, with most of their expression profiles studied in numerous cancer types (Scanlan *et al*, 2004). The function of the majority of the CTAs is still unknown; however, some CTAs are thought to be implicated in the regulation of gene expression and others may control gametogenesis (Old, 2001; Kalejs and Erenpreisa, 2005). The CTAs are attractive targets for developing cancer-specific immunotherapy because of their highly restricted expression in normal tissues and broad expression in a wide range of tumours (Chitale *et al*, 2005).

The human *BORIS* gene maps to chromosome 20q13.2 (Loukinov *et al*, 2002). This chromosome region is often amplified in many cancers and is believed to contain a dominant immortalising or transforming gene(s) (Tanner *et al*, 1994; Cuthill *et al*, 1999). Recent reports show that BORIS is a downstream regulator of cancer–testis genes: expression of BORIS in normal cells leads to derepression of cancer–testis genes *MAGE-A1*, *NY-ESO-1* and others (Hong *et al*, 2005; Vatolin *et al*, 2005).

CTCF and BORIS are expressed in a mutually exclusive manner during male germ-line development (Klenova *et al*, 2002; Loukinov *et al*, 2002); thus, suggesting that BORIS may be important for epigenetic reprogramming occurring during development in these cells. Indeed, BORIS has been implicated in the initiation of a series of methylation events at the imprinting control regions, in the vicinity of the CTCF-/BORIS-binding sites (Jelinic *et al*, 2006), which may be significant for cancer development (Jelinic and Shaw, 2007).

As BORIS is not normally expressed in females, we asked whether BORIS would be present in breast tumours and if so, whether it would have characteristics of a cancer biomarker. In this study, we demonstrate that BORIS protein indeed appears in all breast cancer cell lines tested and in 70.7% of breast tumours. We also identified positive correlations between the levels of BORIS and progesterone receptor (PR) and oestrogen receptor (ER) in breast tumours.

## MATERIALS AND METHODS

### Patients and controls

Primary human tumour breast tissues together with paired normal peripheral tissues were collected during surgery from patients treated at Colchester General Hospital (Essex, UK), with written consent taken before surgery. The study was approved by the Local Ethics Committee (reference number LREC MH363). Normal breast tissue samples were collected after reduction surgery. Tissue specimens were visually examined by an experienced pathologist as fresh material, tumour tissues were selected by conventional pathological criteria and the histopathology was further confirmed by microscopic examination. The paired normal peripheral tissues were removed during macroscopic examination of the tumour by the pathologist. The samples were immediately frozen and stored at –80°C. Histology of the tissues is described in Table 1. The following patient information was provided: tumour stage, tumour grade, tumour size, ER status, PR status, HER-2 receptor status, lymph node metastasis, preoperative and post-operative chemotherapy, menopausal state and patient's age.

### Procedures

#### Cell lines

A panel of 18 breast cell lines of different origins composed of non-malignant and malignant human breast cells was obtained from M O'Hare and B Gusterson. The two non-malignant cell lines were HBL100 (Gaffney, 1982) and HB4a. The HB4a cells were established from normal breast luminal cells immortalised with SV40 T-antigen (Stamps *et al*, 1994). Malignant breast cell lines included six ER-positive (ER+) cells originating from human breast carcinomas (T47D, MCF7, BT474, CAMA1, ZR75-1 and ZR75-30). Ten ER-negative breast cancer cell lines (ER−) contained HMT3522 (a cell line derived from fibrocystic breast tissue); adherent carcinomas MDA-MB-175, MDA-MB-157, MDA-MB-231, MDA-MB-435, MDA-MB-453, MDA-MB-468, SKBR5 and SKBR7; and non-adherent carcinoma DU4475. All breast cancer cell lines were maintained in RPMI-1640 medium supplemented with HEPES, GlutaMAX and sodium bicarbonate, 50 *μ*g ml^−1^ gentamicin, 10% fetal calf serum (all from Life Technologies, Paisley, UK). The HB4a cells were maintained in the same medium with the addition of 5 *μ*g ml^−1^ of insulin and 5 *μ*g ml^−1^ of hydrocortisone (both from Sigma, Poole, UK). Breast cell lines were grown at 37°C and 5% CO_2_. Transformed human epithelial kidney cells, 293T, were maintained in DMEM supplemented with 10% fetal calf serum and 50 *μ*g ml^−1^ gentamicin.

### Expression vectors and transient transfections

The BORIS expression vector (pCMV6–BORIS) used in transient transfections was described previously (Loukinov *et al*, 2002). The pGL2-hPR (or pPRLuc) reporter construct was a kind gift from I De Vivo. It contains the 1536-bp fragment (−711 to +825) of the promoter of the *PR* gene fused with the luciferase reporter gene in pGL2-basic plasmid (De Vivo *et al*, 2002; Huggins *et al*, 2006). The pER3500-230L (or pERLuc) reporter construct was a kind gift from RJ Weigel. It contains the 3730-bp fragment (−3500 to +230) of the promoter of the *ER* gene fused with the luciferase reporter gene in pGL2-basic plasmid (deConinck *et al*, 1995). Transient transfections were performed using a calcium phosphate transfection protocol (Chen and Okayama, 1987).

### Luciferase assay

Following transfection, cells were harvested and assayed using the Luciferase Assay System according to the manufacturer's instructions (Promega, Southampton, UK). Luminescence was measured using a Labsystems Luminoskan luminometer (Life Sciences, Runcorn, UK). To normalise for cell number and transfection efficiency, 0.25 *μ*g *β*-galactosidase marker gene plasmid (pCH110) was included per well in the transfection solution. Mean and s.d. were calculated from the results of three experiments performed in triplicate.

### Primary breast cell culture

To obtain primary cells from fresh normal tissues, we essentially used a method described earlier (Clarke *et al*, 1994) with modifications. In brief, tissues were cut into small pieces (1 mm^3^) and incubated for 2 h on a rotating wheel at 37°C in the presence of 2.5 mg ml^−1^ of collagenase type I from *Clostridium histolyticum* (Sigma). After filtration through a 100-*μ*m sieve (Falcon, Fisher Scientific, Loughborough, UK), the suspension was centrifuged at 1000 **g** for 10 min. Pellets were re-suspended in fresh Leibovitz medium and centrifuged at 1000 **g** for 10 min. The resulting pellet was washed three times with the filter-sterilised washing buffer (1 mM EDTA, 1% BSA in PBS). The final cell pellet was re-suspended in 5 ml of MEBM (mammary epithelium basal medium) (Cambrex, now Lonza, Wokingham, UK), subsequently filtered through a 100-*μ*m and then a 40-*μ*m sieve (Falcon, Fisher Scientific, Loughborough, UK); after that cells were plated in MEBM without sodium bicarbonate supplemented with the MEGM SingleQuots (Cambrex) and fungizone, amphotericin B (0.25 *μ*g ml^−1^) (all from Gibco, Invitrogen, Paisley, UK).

### RNA isolation, reverse transcription and real-time PCR analysis

Total RNA was isolated from tissues using a TRIZOL Reagent (Invitrogen, Carlsbad, CA, USA) according to the manufacturer's instruction. The isolated RNA was quality tested using the Agilent Bioanalyzer 2100. RNA was then converted to cDNA using random primers and Thermoscript reverse transcriptase (Invitrogen). Real-time RT–PCR analysis was performed as described previously (Vatolin *et al*, 2005) using the Applied Biosystems 7900HT Fast Real-Time PCR System. BORIS primers/probe sequences were as follows: 5′-CCCATTGTGCCACCATCA-3′ (forward); 5′-AGCATGCAAGTTGCGCATAT-3′ (reverse); 6FAM-ACGGAAAAGCGACCTAC-MGB (BORIS probe). Glyceraldehyde-3-phosphate dehydrogenase (GAPDH) primers/probe mixture was purchased as Predeveloped Assay (Applied Biosystems, Foster City, CA, USA). Levels of *BORIS mRNA* were expressed in relative copy numbers normalised against the housekeeping gene *GAPDH* (comparative CT method).

### The chicken anti-BORIS antibody

The chicken anti-BORIS antibody was generated and characterized as described previously (Loukinov *et al*, 2002). In the present study, this antibody was further assessed for its specificity in a series of additional assays as described in the Supplementary Figure 1 published online.

### Western blot analysis

For western blot analysis, lysates from cells were prepared according to Klenova *et al* (1993) with modifications. Lysates from breast tissues were prepared as follows. Tissue was homogenized in the lysis buffer at the ratio 500 mm^3^ tissue/100 *μ*l buffer. The buffer was composed of 20 mM Tris-buffered with HEPES pH 8.0/2 mM EDTA/0.5 M NaCl/0.5% Na deoxycholate/0.5% Triton X-100/0.25 M sucrose; 50 mM 2-ME, 250 *μ*M PMSF and 1 *μ*M pepstatin (10 *μ*l/10 ml) were added to the buffer immediately before use. The homogenate was kept on ice for 30 min, filtered through gauze and centrifuged for 15 min, at +4°C and 13 000 r.p.m. Samples containing high concentration of lipids were additionally precipitated with acetone. For this purpose, an equal volume of acetone was added to the supernatant, and the solution was mixed, kept at −20°C for 1 h and then centrifuged for 10 min at +4°C and 13 000 r.p.m. The supernatant was discarded, pellet dried at room temperature and re-suspended in SDS-loading/lysis buffer. Western blot assay was conducted as described previously with the anti-BORIS or anti-*α*-tubulin (Sigma) antibodies (Loukinov *et al*, 2002). Detection was performed with enhanced chemiluminescence reagent (Amersham Biosciences, now GE Healthcare, Buckingham, UK) according to the manufacturer's instructions. Quantification of the bands was performed by using the Image J software (http://rsb.info.nih.gov/ij/), and values were obtained from the ratios CTCF/*α*-tubulin.

### Immunostaining

Immunohistochemical analysis was performed by staining with the Vectastain Elite ABC standard kit (Vector Laboratories, Peterborough, UK) as suggested by the manufacturer. The dilution of the chicken anti-BORIS antibody applied to the sections was 1 : 50 and the secondary goat biotinylated anti-chicken antibody (Vector) was used at 1 : 150 dilution. The sections were then counterstained with haematoxylin only or with haematoxylin and eosin. The immunological staining was evaluated by using the immunoreactivity score (IRS) as described previously (Beck *et al*, 1995). In brief, the percentage of CTCF-positive cells was divided into four categories: (1) <10%, (2) 11–50%, (3) 51–80% and (4) >80%, whereas the staining intensity was given a scale from 0 (no detectable immunostaining) to 3 (strong immunostaining). The IRS (0–12) was then calculated by multiplying the score values. Scoring was performed in a blinded fashion by two independent scorers, with each slide read twice; the results in the Table 1 represent the average score for each sample. The IRS was considered significant when the values were more than 1.

### Statistical analysis

Statistical analysis was carried out using unpaired Student's *t*-test. A significant value was detected when the probability was below the 5% confidence level (*P*<0.05).

## RESULTS

### Analysis of BORIS expression in breast cell lines

**Figure 1:**
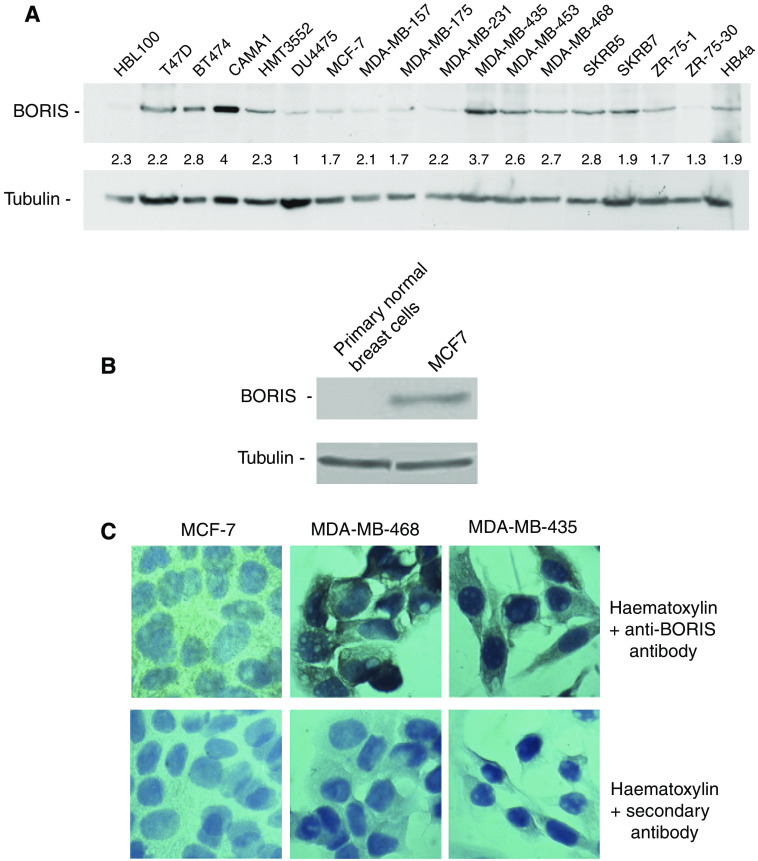
BORIS protein is present in breast cancer cell lines. (**A**) Western blot analysis of BORIS protein levels in human breast cell lines. Cellular extracts were prepared from 5 × 10^6^ cells and equal amounts (20 *μ*g) of total protein were loaded onto SDS–PAGE. Samples were electrophoretically separated, blotted and probed with the anti-BORIS antibody. The membrane was re-probed with the anti-*α*-tubulin antibody, which served as an internal control for protein loading. The images were quantified using the Image J software. The ratios of the intensity of the BORIS bands over the intensity of the corresponding *α*-tubulin bands were determined and expressed as fold change relative to the lowest BORIS/*α*-tubulin ratio found in DU4475 (designated as 1.0). Numbers below each BORIS lane show these results. (**B**) Western blot analysis of primary breast cells compared to MCF-7 cells. Cellular extracts were prepared from 5 × 10^6^ cells and equal amounts (40 *μ*g) of total protein were loaded onto SDS–PAGE. Samples were electrophoretically separated, blotted and probed with the anti-BORIS antibody. The membrane was re-probed with the anti-*α*-tubulin antibody, which served as an internal control for protein loading. (**C**) Immunostaining of BORIS in breast cell lines with low and higher expression levels of BORIS. Cells were grown in eight-well chamber slides, fixed with 4% formaldehyde followed by immunohistochemistry protocol as described under the Materials and Methods section. Cells immunostained with the anti-BORIS antibody are shown in the upper panel. Panels below demonstrate background staining with haematoxylin plus secondary antibodies only. Images were taken at × 100 magnification.

We first investigated the levels of BORIS protein in a panel of breast cancer cell lines composed of two non-malignant (HBL100 and HB4a) and 16 malignant cell lines. Western blot analysis revealed that all cell lines contained the 85-kDa band characteristic for BORIS (Figure 1A). However, the levels of BORIS were different in the cell lines tested: the lowest were observed in the cancer cell lines DU4475 and ZR-75-30; the highest levels were detected in CAMA1 and MDA-MB-435 (Figure 1A). No BORIS was detected in primary breast cells obtained from breast reduction (BR) tissues (Figure 1B).

We then performed immunohistochemical staining with anti-BORIS antibody in a small panel of breast cancer cell lines to confirm BORIS expression. Consistent with the results of the western analysis, low levels of BORIS were detected in the breast cancer cell line MCF-7, whereas the increased levels of BORIS, particularly in the cytoplasm, was observed in MDA-MB-468 and MDA-MB-435 (Figure 1C, upper panel). Although the levels of BORIS in MDA-MB-468 were lower than in MDA-MB-435 in the western assay, they seemed higher in the immunostaining. The explanation for this is given in the Discussion section. No staining was detected with the secondary antibody only (Figure 1C, lower panel). From these data, we conclude that BORIS protein is absent in normal breast cells; however, it is present at variable levels in all breast cancer cell lines, both in the nucleus and in the cytoplasm, in agreement with the earlier report (Loukinov *et al*, 2002).

### Analysis of BORIS expression in breast tumour tissues

**Figure 2:**
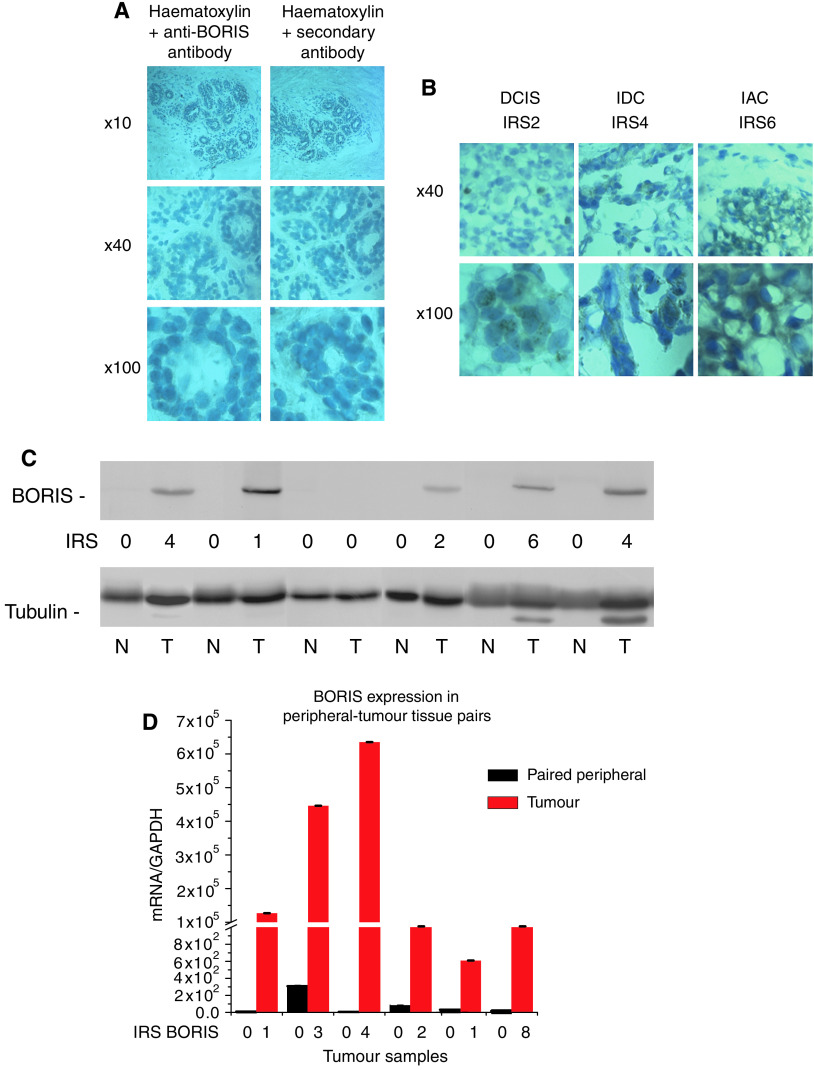
BORIS expression in normal and tumour breast tissues. (**A**) BORIS expression in the BR mammoplasty tissue. Frozen BR tissue was sectioned and immunostained with the anti-BORIS antibody as described under the Materials and Methods section. Left panel, primary and secondary antibody; right panel, secondary antibody only. Images were taken at × 10, × 40 and × 100 magnification. (**B**) BORIS expression in breast tumours with different IRS. Frozen breast tumour tissues were sectioned and immunostained with the anti-BORIS antibody as described under the Materials and Methods section. Images were taken at × 40 (upper panel) and × 100 (lower panel) magnification. DCIS=ductal carcinoma *in situ*; IDC=invasive ductal carcinoma; IAC=invasive adenocarcinoma. (**C**) Western blot analyses of BORIS in selected breast tissues with different IRS and the corresponding PP tissues. Extracts from tissues were prepared as described under the Materials and Methods section, total protein concentration was determined for each sample and equal amount (20 *μ*g) of total protein was loaded onto SDS–PAGE. Samples were electrophoretically separated, blotted and probed with the anti-BORIS antibody. The same membrane was stripped and re-probed with the *α*-tubulin antibody (loading control). (**D**) Real-time RT–PCR analysis of *BORIS* mRNA in selected breast tumours with different IRS paired with the corresponding peripheral tissue. Levels of *BORIS* mRNA were calculated using comparative *C*_t_ method (ΔΔ*C*_t_) and normalised to *GAPDH* levels.

**Figure 3:**
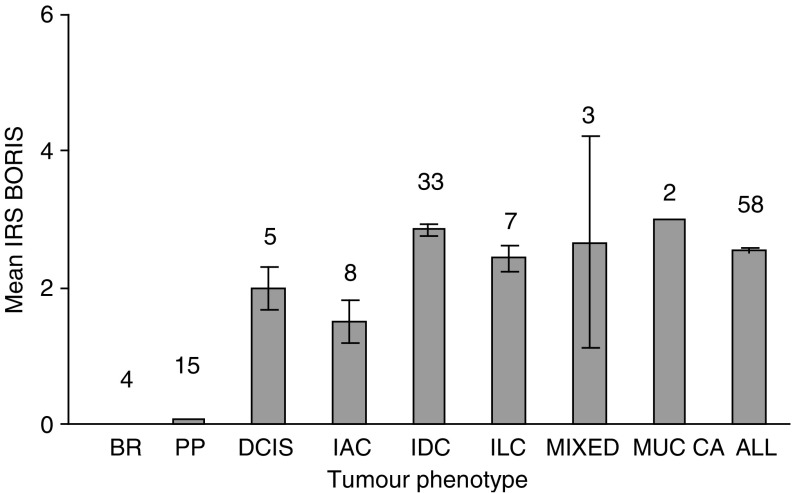
Comparison between BORIS levels in BR tissues, PP tissues and tumours with different phenotypes. The data are shown as mean IRS for BORIS with s.e. BR=breast reduction tissues; PP=paired peripheral breast tissues, DCIS=ductal carcinoma *in situ*; IAC=invasive adenocarcinoma; IDC=invasive ductal carcinoma; ILC=invasive lobular carcinoma; MIXED=tumours with mixed phenotypes; MUC CA=mucinous carcinoma; ALL=all phenotype group.

We then assessed BORIS expression in four BR tissues. The immunohistochemical staining for BORIS was negative in all these tissues (a typical example is shown in Figure 2A and data presented in Table 1 and Figure 3). Next, we used paired peripheral (PP) breast tissues obtained from the same patient with breast tumour. We carried out immunohistochemical staining for BORIS on 15 available PP tissues. The staining revealed that only one sample was very weakly positive for BORIS (IRS<1). The average IRS value for this group was 0.067±0.067. These results are presented in Table 1 and Figure 3. We then evaluated BORIS expression in a panel of 58 primary breast tumours, which consisted of tumours with different phenotypes: five ductal carcinomas *in situ* (DCIS), eight invasive adenocarcinomas (IAC), 33 invasive ductal carcinomas (IDC), seven invasive lobular carcinomas (ILC), three tumours with mixed phenotypes (MIXED) and two mucinous carcinomas (MUC CA). These results are presented in Table 1 and Figure 3. As determined by immunohistochemical staining, BORIS was detected in 70.7% (41 of 58 samples), with the average IRS=2.53±0.04 (‘all phenotype group’).

The IRS values for BORIS in different groups were as follows: 2±0.316 in DCIS; 1.5±0.298 in IAC; 2.85±0.079 in IDC; 2.43±0.199 in ILC; 2.67±1.539 in mixed tumours and 3±0 in mucinous carcinomas. Using an unpaired Student's *t*-test, we confirmed that the difference was significant for the following groups: DCIS, *P*⩽0.05; IAC, *P*⩽0.05; IDC, *P*⩽0.0002; ILC, *P*⩽0.004, and ‘all tumour’, *P*⩽0.003. There were insufficient numbers of samples in the mixed tumours and mucinous carcinomas for statistical analysis.

Figure 2B represents typical examples of immunohistochemical staining for BORIS in breast tumour tissues with different IRS. Similarly to the breast cell lines, the distribution of BORIS is both nuclear and cytoplasmic. The staining also reveals heterogeneity of different tissue specimens for BORIS. Thus, different proportions of BORIS-positive tumour cells and different degrees of BORIS expression can be observed in the specimens (Figure 2B and Table 1).

We also performed western analysis and real-time RT–PCR on randomly selected tumour samples with different IRS and the corresponding PP tissues. All PP tissues tested by western analysis were negative for BORIS, whereas BORIS was present in all samples except the specimen with the IRS BORIS=0 (Figure 2C). However, there was no correlation between the IRS values and the intensity of the band. We also observed higher levels of *BORIS* mRNA in the tumours compared with the PP tissues (Figure 2D); no relationship between the IRS values and levels of *BORIS* mRNA were found in this case. Possible explanations for the absence of such correlations are given in the Discussion section.

### High BORIS levels in breast tumours correlate with high levels of the ER and PR

We also investigated a possible correlation between BORIS levels in breast tumour tissues and clinical/pathological parameters: tumour stage, tumour grade, tumour size, ER status, PR status, HER-2 receptor status, lymph node metastasis, preoperative and post-operative chemotherapy, menopausal state and patient's age. No correlation was found between BORIS levels and these parameters with the exception of ER and PR.

**Figure 4:**
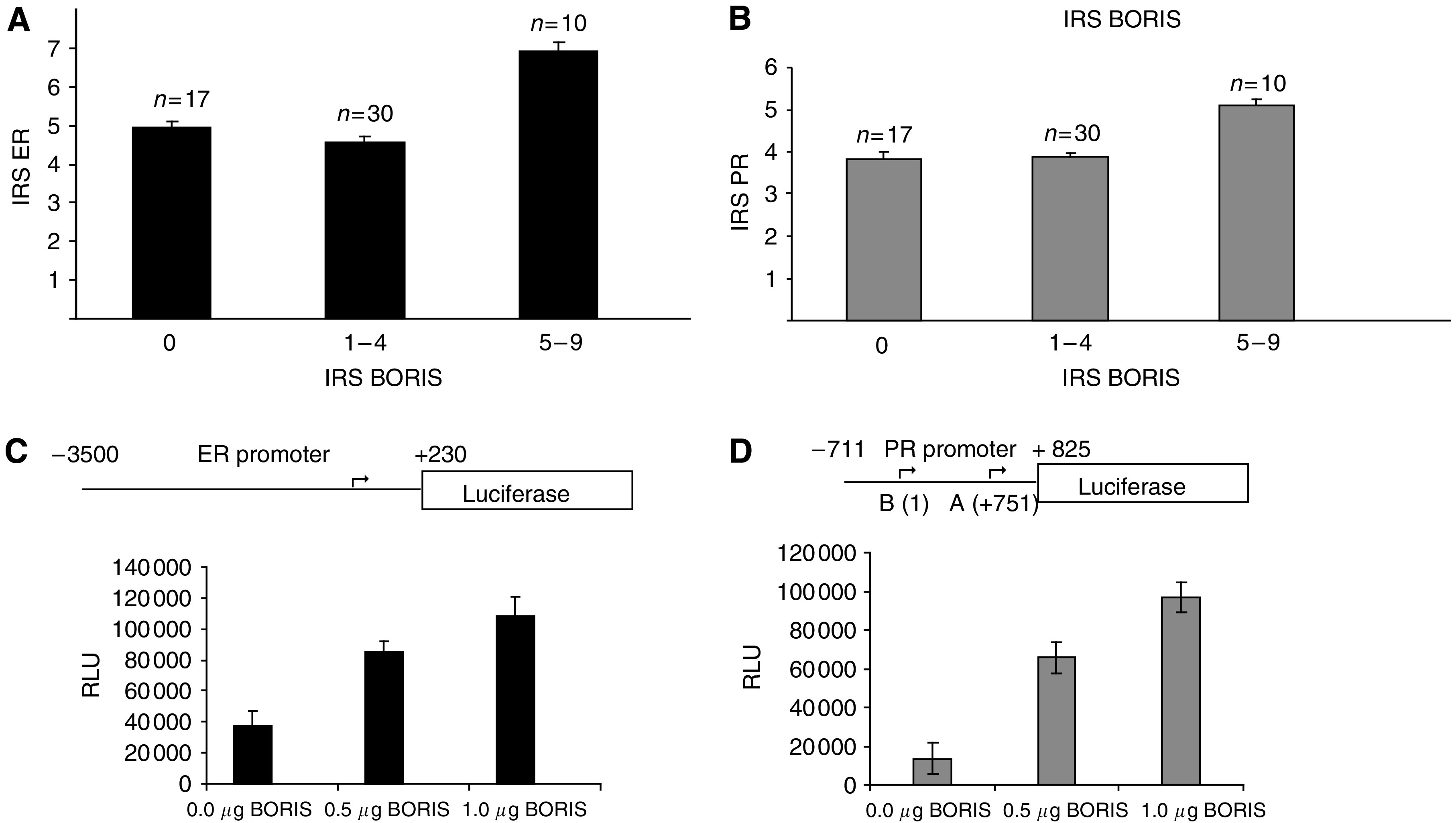
High levels of BORIS correlate with high levels of ER and PR. (**A** and **B**) Comparison between BORIS and ER (**A**) and BORIS and PR (**B**) levels in breast tumours. Three groups with the following levels of BORIS were analysed: IRS=0, IRS=1–4 and IRS=5–9. BORIS levels were assessed in the entire tumour group; all phenotypes were included in the analysis. The mean IRS value for ER and PR are shown with the standard error. (**C** and **D**) BORIS activates transcription from the promoters of the *ER* and *PR* genes in reporter gene assays. For transient transfections, 293T cells (1.5 × 10^5^) were plated in 12-well dishes; 1 *μ*g of the reporter constructs pERLuc (**C**) and pPRLuc (**D**) containing promoters of *ER* and *PR* genes fused to the luciferase reporter gene were then co-transfected with the increasing concentration of pCMV6-BORIS (shown on the graphs). The diagrams of the reporter constructs are presented in the upper parts of the panels. Dark line depicts sequences derived from the promoter regions; arrows indicate the transcription start (one site in the *ER* gene and two sites, A and B, in the *PR* gene). Numbers correspond to the distance from the transcription start site. Forty-eight hours post-transfection cells were harvested and assayed for luciferase activity as described under the Materials and Methods section. Bars represent luciferase activity in relative luciferase units (RLU). Each bar shows an average of three experiments performed in triplicate. Error bars indicate s.d.

To compare BORIS levels with ER and PR, we divided all tumours into three groups according to the BORIS values: IRS=0, IRS=1–4 and IRS=5–9. The mean IRS for ER in the groups with BORIS IRS=0 and BORIS IRS=1–4 did not differ (4.94±0.18 *vs* 4.56±0.17, respectively) (Figure 4A). Similarly, there was no difference between these for IRS PR (3.82±0.18 *vs* 3.87±0.1, respectively) (Figure 4B). However, we observed higher levels of ER and PR (6.9±0.26 and 5.1±0.14, respectively) in the group with higher levels of BORIS (IRS=5–9) (Figure 4A and B).

### BORIS activates promoters of the *PR* and *ER* genes in the reporter assays in breast and non-breast cells

BORIS has features of a transcriptional regulator and therefore, we asked whether BORIS could regulate the promoters of *PR* and *ER* genes. To investigate this, we employed luciferase reporter gene constructs containing the promoter regions of *PR* (De Vivo *et al*, 2002) and *ER* (deConinck *et al*, 1995) genes inserted upstream of the luciferase reporter gene (Figure 4C and D). A series of standard co-transfection assays were performed in human epithelial kidney cells, 293T, with these reporter constructs and a vector expressing the full-length BORIS (pCMV6-BORIS). We found that BORIS progressively activates both promoters in these cells (Figure 4C and D); thus, suggesting that BORIS can be a positive regulator of transcription of both genes. Similar results were also observed in the breast cancer cell line, ZR-75-1 (Supplementary Figure 2). On the other hand, there was no effect from the increasing concentrations of BORIS on the basic reporter luciferase construct, pGL2, in both cell lines (Supplementary Figure 3). These results corroborate our previous observations of correlation between high BORIS levels in breast tumours and high levels of the ER and PR (Figure 4A and B).

## DISCUSSION

The aim of this study was to investigate BORIS expression in breast cancer cells and tumours. Although the antibody used in this investigation was previously characterized for their specificity for BORIS (Loukinov *et al*, 2002), we performed additional experiments to confirm these observations (Supplementary Figure 1). In these experiments, the polyclonal chicken anti-BORIS antibody was shown to be specific by the following criteria: (1) intensity of the immunohistochemical staining of the breast tumour tissue was significantly reduced after preincubation of the antibody with the blocking peptide, (2) intensity of the immunofluorescent staining of the cells in culture was significantly reduced after preincubation of the antibody with the blocking peptide, (3) western analysis of the N-terminal domain of BORIS expressed in *E. coli* and the protein produced from the full-length BORIS cDNA in the eukaryotic-expressing vector in 293T cells demonstrated the presence of the recombinant products, recognised by the anti-BORIS antibody and (4) preparative immunoprecipitation of the cell lysates with the anti-BORIS antibody and subsequent analysis of the 85-kDa band characteristic for BORIS by mass-spectrometry (MS/MS sequencing), revealed the presence of peptides matching BORIS in this band.

Using this antibody, we found that BORIS was expressed at different levels in all 18 breast cancer cell lines used in the experiments. It is, however, difficult to correlate the levels of BORIS detected by western analysis and immunohistochemical staining of the same cells (e.g. MDA-MB-435 and MDA-MB-468, Figure 1A and C). Thus, the apparently denser accumulation of staining in MDA-MB-468 cells as seen by the immunohistochemistry can be due to different cell morphology of MDA-MB-468 (small cytoplasm, more rounded cell shape).

As BORIS was detected in 70.7% of breast tumours, the presence of BORIS in 100% cell lines may indicate that the selective pressure *in vivo* on some tumours may be absent *in vitro*. The availability of the component(s) in the growth media that may be important for BORIS activation could be a factor; access to such component(s) may be restricted in the microenvironment of some tumours. Given the complex nature of BORIS regulation at the transcriptional level it may be possible that different promoters are involved in the control of BORIS expression in cultured cells and tumours; thus, providing different molecular mechanisms of BORIS activation *in vivo* and *in vitro* (Renaud *et al*, 2007) (see below).

There was no obvious correlation between the amount of BORIS in non-malignant and malignant cells, which suggests that BORIS may not play a key role in the establishment of the malignant phenotype. This is supported by the lack of correlation between BORIS levels in breast tumours and lymph node metastasis, which is used in clinical practice as an indicator of the malignant breast cancer (Tobler and Detmar, 2006; Eccles *et al*, 2007). On the other hand, BORIS was not detected in primary normal breast cells, which suggests that BORIS is likely to be associated with the immortalised and malignant cells.

The immunohistochemical staining revealed that the levels of BORIS protein were significantly higher in all breast tumours compared with normal and PP tissues; 70.7% of breast tumours were BORIS-positive. All normal breast tissues from BR specimens were negative for BORIS. Only 1 tissue out of 15 from the group of the PP tissues was very weakly positive; we explain this by influence of the tumour on normal tissue, which may take place in some cases. Such occurrences are well documented in the literature for mRNA/proteins for both CTA and non-CTA genes (Janz *et al*, 2002; Zhao *et al*, 2004).

In our investigation, the levels of the *BORIS* mRNA were considerably higher in breast tumour tissues compared with the PP tissues. However, no correlation was observed between the levels of *BORIS* mRNA and protein (Figure 2D). Lack of coordinated expression between mRNA and protein is not unusual – poor correlation between expression of an mRNA and the corresponding protein is now generally acknowledged and accepted (Abbott, 1999). Genes showing no obvious direct link between expression of mRNA and corresponding protein in various cell lines and tumours are described in the literature and include p27^Kip^ (Ciaparrone *et al*, 1998), ERs and PRs (Tong *et al*, 1999). The heterogenous nature of breast tumour tissues may also account for this discrepancy as different parts of the tissues are used for extraction of protein and mRNA. The latter may also be the reason for the lack of good correlation between the immunohistochemical staining (IRS for BORIS) and western analysis data (Figure 2A and C).

We noted the heterogenous pattern of BORIS staining in breast tumour tissues, showing single BORIS-positive cells or clusters of positive cells, with varying staining intensity. This may explain the fact that the IRS for BORIS has not reached the values higher than 9. Such pattern of expression is common to CTA, examples include distribution of MAGE antigens in various tumours (Jungbluth *et al*, 2000a, 2000b).

Other CTA have also been detected in breast tumours. Although some CTA can be detected in breast tumours of different type, other CTA are observed only in particular breast tumours. Thus, XAGE-1 was observed in invasive lobular as well as ductal carcinoma (Egland *et al*, 2002), and NY-BR-1 was detected in various breast carcinomas (Theurillat *et al*, 2007). MAGE-A was seen frequently in primary ductal breast carcinomas, but not in lobular carcinomas (Otte *et al*, 2001). Almost 81% of the ductal carcinomas have been shown to express PLU-1 (Barrett *et al*, 2002).

Cancer testis antigens are considered to be promising candidates for tumour vaccines because of their immunogenicity and tissue-restricted expression (Scanlan *et al*, 2004; Kalejs and Erenpreisa, 2005). Recent studies using a mouse model revealed that immune response to BORIS can be developed in the organism; and furthermore, such immune response has protective effects against several mouse tumours of different origin (Loukinov *et al*, 2006; Ghochikyan *et al*, 2007). These data indicate that BORIS may be an attractive candidate for the development of the future cancer vaccine. For such a therapy, it will be critical to determine the presence of BORIS in the tumour given that a proportion of breast tumours (29.3%) are BORIS negative.

The incidence of BORIS expression at the protein level (70.7%) observed in the current study corresponds well to the reported findings of *BORIS* mRNA expression in endometrial and uterine tumours (77%) (Risinger *et al*, 2007). The fact that not all tumours contain BORIS may reflect different pathways of evolution in different tumours. Another possibility may be the presence in the tissues of the alternative forms of BORIS not detected by the existing antibodies. Indeed, recently 25 full-length and partial splice variants of BORIS messages coding for 17 different proteins have been cloned and submitted to the GenBank (E Pugacheva *et al*, manuscript in preparation). Six full-length BORIS splice isoforms (GenBank accession nos. DQ778125, DQ778124, DQ778126, DQ778129, DQ778128 and DQ778127) and two partial BORIS cDNA isoforms (GenBank accession nos. DQ778131 and DQ778130) do not have the exon-1 sequence for the peptide recognised by the affinity-purified chicken anti-BORIS antibody (Loukinov *et al*, 2002), which was used in the present study for staining breast tissue sections. Therefore, it is likely that results obtained by the immunostaining with the chicken anti-BORIS antibody may underestimate the actual frequency of presence of BORIS in breast cancers if splice variants undetectable by this antibody are expressed.

In this investigation, we also assessed the clinical significance of BORIS in breast tumours and found that high BORIS levels correlated with high levels of PR and ER. Both oestrogen and progesterone are important hormones in mammary development in humans stimulating cell growth, proliferation and differentiation (Barron *et al*, 1997; Humphreys *et al*, 1997; Anderson, 2002). Moreover, both hormones have been demonstrated to promote breast tumorigenesis (Medina, 2005; Montero Girard *et al*, 2007). It is conceivable that BORIS may stimulate production of both PR and ER, which in turn may support tumour progression. Further experiments confirmed that BORIS could activate promoters of both *ER* and *PR* genes in the reporter assays in cells of breast and non-breast origin; thus, pointing at the possible molecular mechanism of direct transcriptional regulation of these genes. Inspection of the promoter regions of the *ER* and *PR* genes tested in the reporter assays indeed revealed potential-binding sites for CTCF/BORIS (Supplementary Figures 4 and 5). However, further investigations will need to be carried out to confirm direct binding of BORIS to these sites and clarify the role of BORIS in the regulation of *ER* and *PR* genes.

The association of BORIS with immortalised and malignant cells indicates that BORIS may be important in the establishment and maintenance of cell proliferation. This may be linked to the original BORIS function, regulation of gametogenesis, which BORIS is likely to coordinate with other CTA family members (Old, 2001). The appearance of common CTA (including BORIS) during gametogenesis and tumorigenesis prompts the hypothesis that induction of the gametogenetic programme in somatic cells may be associated with tumour development (Old, 2001; Kalejs and Erenpreisa, 2005). As BORIS seems to function as an upstream regulator for several CTA (Hong *et al*, 2005; Vatolin *et al*, 2005), it is tempting to speculate that BORIS could be the ‘master switch’ in the process of cell reprogramming, which can guide the epigenetic machinery to a set of target genes eventually leading to their activation. It is important to note that BORIS may also act as an activator of genes responsible for proliferation in particular tissues, such as *ER* and *PR* genes in mammary glands.

An intriguing question is why and how BORIS itself is activated. Recent reports reveal that both genetic and epigenetic mechanisms are likely to be implicated in this process. Thus, DNA methylation, functional p53 and CTCF play an important role in the negative regulation of the promoters of the *BORIS* gene (Renaud *et al*, 2007). Demethylation of DNA, knockout of CTCF and absence of functional p53 can lead to strong activation of BORIS. Selective utilisation of different promoters (Renaud *et al*, 2007) and alternative splicing (Pugacheva *et al*, manuscript in preparation) are also likely to contribute to regulation of BORIS expression in different cell types. The research efforts of several laboratories are currently focused on uncovering the details of the molecular mechanisms of activation and regulation of BORIS.

## Figures and Tables

**Table 1:** Assessment of the breast tissues for BORIS using immunohistochemical staining with the anti-BORIS antibody

**No**	**Diagnosis**	**Percentage of positive cells (group)**	**Staining intensity**	**IRS tissues**
BR1	NBr	0	0	0
BR2	NBr	0	0	0
BR3	NBr	0	0	0
BR4	NBr	0	0	0
PP3	PP	0	0	0
PP6	PP	<1	<1	<1
PP9	PP	0	0	0
PP11	PP	0	0	0
PP17	PP	0	0	0
PP19	PP	0	0	0
PP23	PP	0	0	0
PP28	PP	0	0	0
PP30	PP	0	0	0
PP33	PP	0	0	0
PP42	PP	0	0	0
PP58	PP	0	0	0
PP71	PP	0	0	0
PP83	PP	0	0	0
PP89	PP	0	0	0
52	DCIS	2	2	4
58	DCIS	2	1	2
168	DCIS	1	1	1
279	DCIS	1	3	3
445	DCIS	0	0	0
2	IAC	0	0	0
3	IAC	0	0	0
19	IAC	0	0	0
28	IAC	0	0	0
71	IAC	1	1	1
89	IAC	2	3	6
144	IAC	2	1	2
210	IAC	2	1	2
6	IDC	1	2	2
11	IDC	1	1	1
15	IDC	0	0	0
16	IDC	0	0	0
17	IDC	1	2	2
23	IDC	0	0	0
30	IDC	1	1	1
41	IDC	1	1	1
51	IDC	0	0	0
73	IDC	2	2	4
83	IDC	2	1	2
135	IDC	0	0	0
138	IDC	0	0	0
192	IDC	2	1	2
195	IDC	3	2	6
197	IDC	3	1	3
209	IDC	3	2	6
214	IDC	3	2	6
222	IDC	2	2	4
228	IDC	3	2	6
229	IDC	2	1	2
251	IDC	3	3	9
258	IDC	0	0	0
281	IDC	3	2	6
290	IDC	2	2	4
292	IDC	4	2	8
309	IDC	2	1	2
326	IDC	2	2	4
328	IDC	2	2	4
352	IDC	0	0	0
393	IDC	0	0	0
428	IDC	3	2	6
453	IDC	3	1	3
265	ILC	1	2	2
332	ILC	2	1	2
333	ILC	2	2	4
357	ILC	0	0	0
413	ILC	3	1	3
417	ILC	2	1	2
446	ILC	2	2	4
9	MIXED	0	0	0
324	MIXED	4	2	8
440	MIXED	0	0	0
33	MUC CA	2	2	4
329	MUC CA	3	1	3

BR=breast reduction tissues; NBr=normal breast reduction tissues; PP=paired peripheral breast tissues; DCIS=ductal carcinoma *in situ*; IAC=invasive adenocarcinoma; IDC=invasive ductal carcinoma; ILC=invasive lobular carcinoma; MIXED=tumours with mixed phenotypes; MUC CA=mucinous carcinoma.

Staining was assessed by the immunoreactivity score (IRS) as described under the Materials and Methods section.
