# BORIS, a paralogue of the transcription factor, CTCF, is aberrantly expressed in breast tumours

**DOI:** 10.1038/sj.bjc.6604255

**Published:** 2008-02-05

**Authors:** V D'Arcy, N Pore, F Docquier, Z K Abdullaev, I Chernukhin, G-X Kita, S Rai, M Smart, D Farrar, S Pack, V Lobanenkov, E Klenova

**Correction to**: *British Journal of Cancer* (2008) **98**, 571–579. doi:10.1038/sj.bjc.6604181

The authors wish to add an acknowledgement to their paper, recently published online (AOP) and in this issue.

The final sentence of the Acknowledgements section should now read (additional acknowledgement in bold).

‘This work was supported by Breast Cancer Campaign (FD and EK), Breast Cancer Research Trust (MS, G-XK and EK), Medical Research Council (DF and EK), Research Promotion Fund from the University of Essex (FD, IC and EK) **and by the Intramural Research Program of the National Institutes of Health, National Institute of Allergy and Infectious Diseases (ZKA, SP and VL)**; VD was supported by a PhD studentship from the BBSRC, UK; NP is Clinical Fellow supported by the Essex Rivers NHS Trust’.

