# Supplementary figures and images for: BORIS, a paralogue of the transcription factor, CTCF, is aberrantly expressed in breast tumours
Source: Br J Cancer. 2008 Jan 15;98(3):571–9. doi: 10.1038/sj.bjc.6604181 (PMC2243163; doi:10.1038/sj.bjc.6604181)

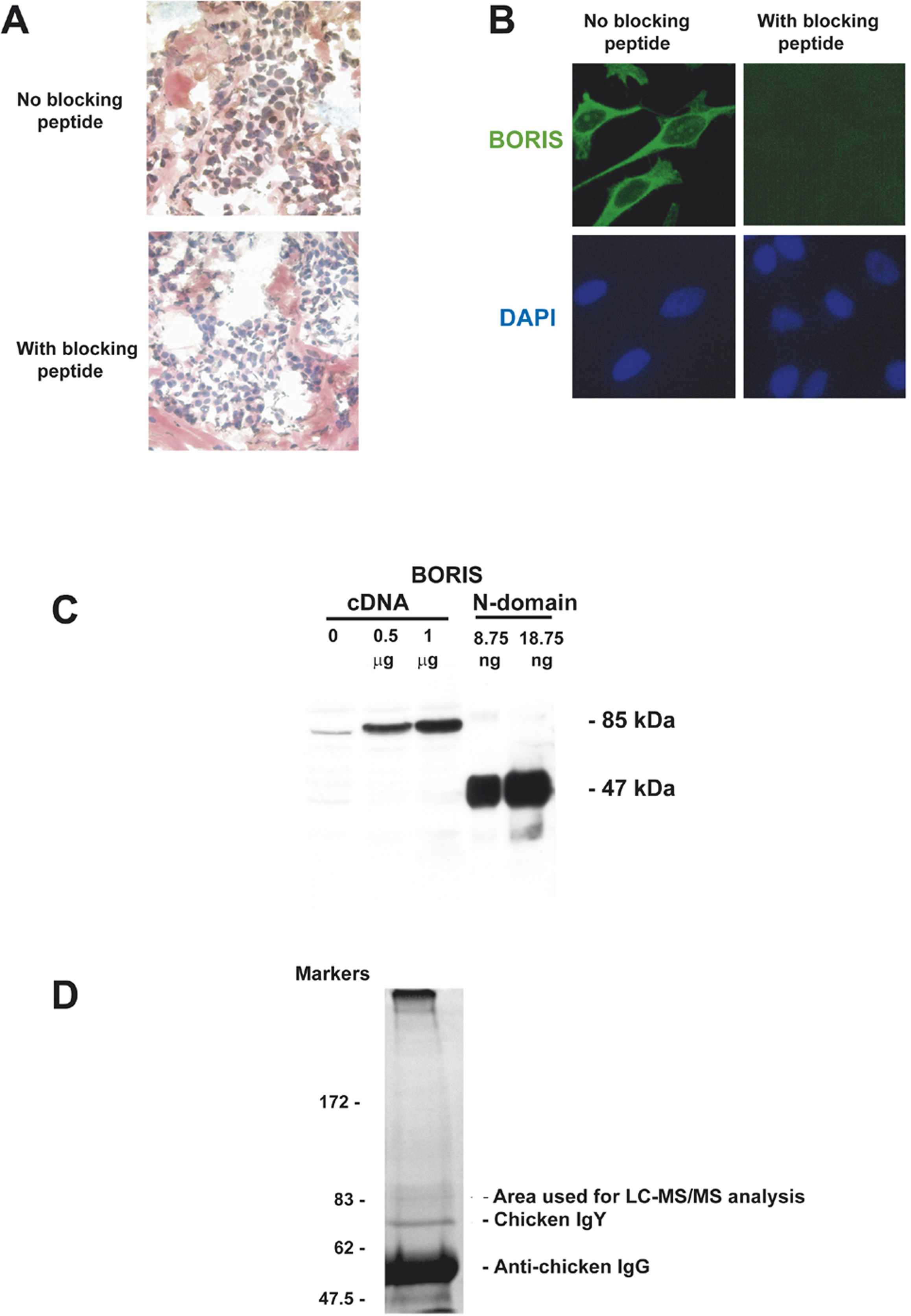

Supplement: Supplementary Figure 1 [file 6604181x1.tif]
