# Supplementary material for: BORIS, a paralogue of the transcription factor, CTCF, is aberrantly expressed in breast tumours
Source: Br J Cancer. 2008 Jan 15;98(3):571–9. doi: 10.1038/sj.bjc.6604181 (PMC2243163; doi:10.1038/sj.bjc.6604181)
Supplement: Supplementary Figure 2 [file 6604181x2.doc]

**A. Reporter pERLuc B. Reporter pPRLuc**

| | 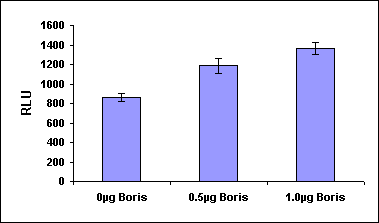 | | --- | |  |  |  | 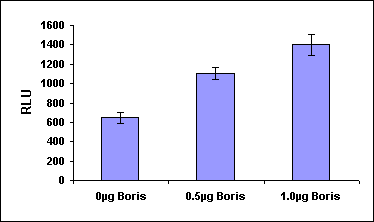 |  |  |  |  |  |  |  |  |
| --- | --- | --- | --- | --- | --- | --- | --- | --- | --- | --- | --- | --- | --- |
|  |  |  |  |  |  |  |  | | | | | |
|  |  |  |  |  |  |  |
|  |  |  |  |  |  |  |
|  |  |  |  |  |  |  |
|  |  |  |  |  |  |  |
|  |  |  |  |  |  |  |
|  |  |  |  |  |  |  |
|  |  |  |  |  |  |  |
|  |  |  |  |  |  |  |
|  |  |  |  |  |  |  |
|  |  |  |  |  |  |  |
|  |  |  |  |  |  |  |
|  |  |  |  |  |  |  |
|  |  |  |  |  |  |  |

**Supplemental Figure 2.** BORIS activates transcription from the promoters of the *ER* and *PR* genes in reporter gene assays in ZR-75-1 cells.

ZR-75-1 cells (2.5x10^5^ ) were plated in 12-well dishes and then transiently co-transfected with 1 μg of the reporter constructs pERLuc (panel A) and pPRLuc (panel B) containing promoters of *ER* and *PR* genes fused to the luciferase reporter gene and the increasing concentration of pCMV6-BORIS (indicated on the graphs). (The maps of the reporter genes are shown in Figure 4). Forty eight hours post-transfection cells were harvested and assayed for luciferase activity as described under Materials and Methods. Bars represent luciferase activity in relative luciferase units (RLU). Each bar shows an average of three experiments performed in triplicate. Error bars indicate standard deviations.
