# Supplementary material for: BORIS, a paralogue of the transcription factor, CTCF, is aberrantly expressed in breast tumours
Source: Br J Cancer. 2008 Jan 15;98(3):571–9. doi: 10.1038/sj.bjc.6604181 (PMC2243163; doi:10.1038/sj.bjc.6604181)
Supplement: Supplementary Figure 3 [file 6604181x3.doc]

A. 293T cells B. ZR-75-1 cells

| 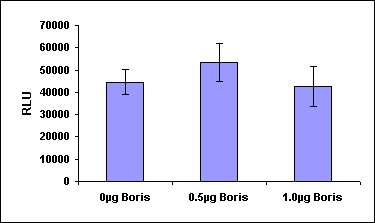 |  |  |  | 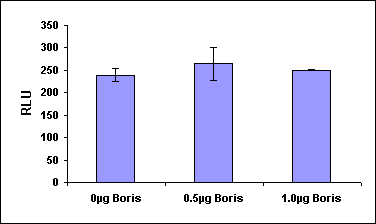 |  |  |  |  |
| --- | --- | --- | --- | --- | --- | --- | --- | --- |
|  |  |  |  |  |  |  |  |  |
|  |  |  |  |  |  |  |  |  |
|  |  |  |  |  |  |  |  |  |
|  |  |  |  |  |  |  |  |  |
|  |  |  |  |  |  |  |  |  |
|  |  |  |  |  |  |  |  |  |
|  |  |  |  |  |  |  |  |  |
|  |  |  |  |  |  |  |  |  |
|  |  |  |  |  |  |  |  |  |
|  |  |  |  |  |  |  |  |  |
|  |  |  |  |  |  |  |  |  |
|  |  |  |  |  |  |  |  |  |
|  |  |  |  |  |  |  |  |  |

**Supplemental Figure 3.** BORIS has no effect on the activity of the basic pGL2- Luciferase vector in reporter gene assays in 293T and ZR-75-1 cells.

293T (1.5x10^5^, panel A) and ZR-75-1 (2.5x10^5^, panel B ) were transiently co-transfected with 1 μg of the basic luciferase reporter construct, pGL2 and the increasing concentration of pCMV6-BORIS (shown on the graphs). Forty eight hours post-transfection cells were harvested and assayed for luciferase activity as described under Materials and Methods. Bars represent luciferase activity in relative luciferase units (RLU). Each bar shows an average of three experiments performed in triplicate. Error bars indicate standard deviations.
