# Supplementary material for: BORIS, a paralogue of the transcription factor, CTCF, is aberrantly expressed in breast tumours
Source: Br J Cancer. 2008 Jan 15;98(3):571–9. doi: 10.1038/sj.bjc.6604181 (PMC2243163; doi:10.1038/sj.bjc.6604181)
Supplement: Supplementary Figure 4 [file 6604181x4.doc]

**Supplemental Figure 4**

**Potential CTCF/BORIS binding sites within the promoter of the *ER* gene**

**A:** CTCF/BORIS consensus sequence as determined by Kim et al (1) is shown as DNA logo. Consensus previously reported by Chao et al (2) and Bell and Felsenfield (3) is shown in black. The “consensus” has key conserved nucleotides C, G, and G and C together

at positions 6, 11, 14, and 16; nucleotides at other positions can vary.

**B.** **Potential CTCF/BORIS binding sites within the promoter of the *ER* gene (-3500+230).** Transcription start site P1 is highlighted in green. Potential eight CTCF/BORIS binding sites are highlighted in grey. The translation start site is underlined.

-3500

GAAGAGAATGCTGG

AGAGAAAGTGGTTAAGAAAACTGCCTTTACTGAACTTCTTGGGCTAACTTTGATTGTAAGTCTCTGAACA

ATCAAAGCCTGTGAGGAGACAGCCAACCTTCTTATTCTTCCTATGTCAATAGTGAACAATTGCAGATCCC

CTTTCCTTTCCTTCTCCTTTCCCCTGTTCCTCTCTCCTCCCTCCCTGAATACTCTTGCTTTTTTCTGGGA

CTGGTCTAGAGCATGGGTGGCCATTGTTGACCTACAGGAGGCACCACTGTCACCAACAAAGGGTAACAGT

CTTTCTTTTCAATATTTATTTATATCCAGTATTTATTTTCAATACTGACTATGGAGAGAGCTCTCCTGTG

CTCAAACACTGCAATACTGGGGGTCTTTCAAAGCACAAAAACATATATTTGCATGATGGCATCATTAACA

TTTTTATGGCTTTCTATTTCTTTTTTGTACTGGTCTCAAGAGCCACTCATAAATCTCTCAGTAACTGCAT

AGTGTCCCAGGGCCAGAGACCGGCCACTCCTGGCATTGTGATTAGAGTCATTTAATATCCAAGGTGGTGA

CTAATGTCTGGCAACAAAGCCTCCATTGGGTGTCATGTGTCCTGGGACCCTGAGCGTGGGCACTCTAGGA

GCACCTCAGTATTGCGTGTTAGTACTATGGCCGAGAGAATAGTTGAGAAAGTGGTCAAGAGGTGGATCCA

TGTGAACGCCACTGGGAAATGAGAGACCTCGTTCCCAATCACGGTCAGTGCAACTCGAAAGCCTAAAATC

AGTTTAAAACAAAGGTATCTACCTTTATCTTATGTTCATATCCTAGGCTTTTAATAATACGTATTTTTCA

CATGTTTACAGAAAGCAGTCAACTGAGCTATTCATGGAAAGGTTTGTGGGTTTGGTTAACGAAGTGGAGG

AGTATTACATTTCAGCTGGAAACACATCCCTAGAATGCCAAAACATTTATTCCAAAGTCTGGTTTCCTGG

TGCAATCGGAGGCATGGCAATGCCTCTGTTCAGAGACTGGGGGCTAGGGCCAGTAAGGCATTTGATCCAC

ATGTATCCCAGAAGGCTTTTATTGTTAAATTATATTCTTTCGGAAAAACCACCCATGTCCTATTTTGTAA

ACTTGATATCCATACACTTTTGACTGGCATTCTATTTTAGCCGTAAGACTATGATTCACAGCAAGCCTGT

TTTTCCTCTTGCTTGGGGTGGCAGCAGAAAGCATAGGGTACTTTCCAGCCTCCAAGGGTAGGGGCAAAGG

GGCTGGGGTTTCTCCTCCCCAGTACAGCTTTCTCTGGCTGTGCCACACTGCTCCCTGTGAGCAGACAGCA

AGTCTCCCCTCACTCCCCACTGCCATTCATCCAGCGCTGTGCAGTAGCCCAGCTGCGTGTCTGCCGGGAG

GGGCTGCCAAGTGCCCTGCCTACTGGCTGCTTCCCGAATCCCTGCCATTCCACGCACAAACACATCCACA

CACTCTCTCTGCCTAGTTCACACACTGAGCCACTCGCACATGCGAGCACATTCCTTCCTTCCTTCTCACT

CTCTCGGCCCTTGACTTCTACAAGCCCATGGAACATTTCTGGAAAGACGTTCTTGATCCAGCAGGGTAGG

CTTGTTTTGATTTCTCTCTCTGTAGCTTTAGCATTTTGAGAAAGCAACTTACCTTTCTGGCTAGTGTCTG

TATCCTAGCAGGGAGATGAGGATTGCTGTTCTCCATGGGGGTATGTGTGTGTCTCCTTTTTCTTTCAGGA

CTTGTAGGATTCTTTGTGCCATTTGCATATAATTTGGCAGGTTCACATTTTTTAAGAGCCCTATGAAGTG

CTTTTTGCATGTGTTTTAAAAAGGCATTTGAAAATTGAAAGTGTGATTTATGGAAATTAAATCATCTGTA

AAAAATTGCTTTGGAAAGTAATGATTGCTGGCCATAAAGGGAAATATCTGCGATGCACCTAATGTGTTTT

TAACCCTTTATTTGCTGACAATCTATAGTCATTAATGCTAAACTCGATTTTGGCTTCAGCTACATTTGCA

TATTGTCCAACAATGGTCTATTTTTGTAAGAATTAGATAAAATGTATACTTGATATAAAATAGTCAAAAA

TGTAACTCTTAGTAACAGTAAGCTTGGCATTTAGATAGACCATGAACACTTCGTCAGATACTCTGTTGGG

TGTTTGGGATAGCAATTAAAACAAAGTATTGATAGTTGTATCAGAGTCTATTAGGCTGCAGCAAAGGAAG

TTTATTCAAAAGTATAAACTATCCAAGATTATAGACGCATGATATACTTCACCTATTTTTTGTCTCCTTA

ATATGTATATATATATATATATATATATATATATATATATATATATATACACATATATGTGTGTGTGTAT

GTGCGTGTGCATGTTTAACTTTTAATTCAGTTAAAAACTTTTTTCTATTTGTTTTTCATCTGGATATTTG

ATTCTGCATATCCTAGCCCAAGTGAACCGAGAAGATCGAGTTGTAGGACTAAAGGATAGACATGCAGAAA

TGCATTTTAAAAATCTGTTAGCTGGACCAGACCGACAATGTAACATAATTGCCAAAGCTTTGGTTCGTGA

CCTGAGGTTATGTTTGGTATGAAAAGGTCACATTTTATATTCAGTTTTCTGAAGTTTTGGTTGCATAACC

AACCTGTGGAAGGCATGAACACCCATGTGCGCCCTAACCAAAGGTTTTTCTGAATCATCCTTCACATGAG

AATTCCTAATGGGACCAAGTACAGTACTGTGGTCCAACATAAACACACAAGTCAGGCTGAGAGAATCTCA

GAAGGTTGTGGAAGGGTCTATCTACTTTGGGAGCATTTTGCAGAGGAAGAAACTGAGGTCCTGGCAGGTT

GCATTCTCCTGATGGCAAAATGCAGCTCTTCCTATATGTATACCCTGAATCTCCGCCCCCTTCCCCTCAG

ATGCCCCCTGTCAGTTCCCCCAGCTGCTAAATATAGCTGTCTGTGGCTGGCTGCGTATGCAACCGCACAC

CCCATTCTATCTGCCCTATCTCGGTTACAGTGTAGTCCTCCCCAGGGTCATCCTATGTACACACTACGTA

TTTCTAGCCAACGAGGAGGGGGAATCAAACAGAAAGAGAGACAAACAGAGATATATCGGAGTCTGGCACG

GGGCACATAAGGCAGCACATTAGAGAAAGCCGGCCCCTGGATCCGTCTTTCGCGTTTATTTTAAGCCCAG

TCTTCCCTGGGCCACCTTTAGCAGATCCTCGTGCGCCCCCGCCCCCTGGCCGTGAAACTCAGCCTCTATC

CAGCAGCGACGACAAGTAAAGTAAAGTTCAGGGAAGCTGCTCTTTGGGATCGCTCCAAATCGAGTTGTGC

CTGGAGTGATGTTTAAGCCAATGTCAGGGCAAGGCAACAGTCCCTGGCCGTCCTCCAGCACCTTTGTAAT

GCATATGAGCTCGGGAGACCAGTACTTAAAGTTGGAGGCCCGGGAGCCCAGGAGCTGGCGGAGGGCGTTC

GTCCTGGGACTGCACTTGCTCCCGTCGGGTCGCCCGGCTTCACCGGACCCGCAGGCTCCCGGGGCAGGGC

CGGGGCCAGAGCTCGCGTGTCGGCGGGACATGCGCTGCGTCGCCTCTAACCTCGGGCTGTGCTCTTTTTC

CAGGTGGCCCGCCGGTTTCTGAGCCTTCTGCCCTGCGGGGACACGGTCTGCACCCTGCCCGCGGCCACGG

ACCATG

+230

**C. The alignment of the potential eight sites:** key nucleotides at positions 6, 11, 14, and 16 are boxed

**GCATGGGTGGCCAT**

**CCATTGGGTGTCAT**

**GCAATCGGAGGCAT**

**CCTGTGGAAGGCAT**

**CCCTCAGATGCCCC**

**TCTGTGGCTGGCTG**

**TCCCTGGCCGTCCT**

**CCCTGCGGGGACAC**

References

1. Kim TH, Abdullaev ZK, Smith AD, et al. Analysis of the vertebrate insulator protein CTCF-binding sites in the human genome. Cell 2007; 128: 1231-45.

2. Chao W, Huynh KD, Spencer RJ, Davidow LS, Lee JT. CTCF, a candidate trans-acting factor for X-inactivation choice. Science 2002; 295: 345-7.

3. Bell AC, Felsenfeld G. Methylation of a CTCF-dependent boundary controls imprinted expression of the Igf2 gene. Nature 2000; 405: 482-5.
