# Supplementary material for: BORIS, a paralogue of the transcription factor, CTCF, is aberrantly expressed in breast tumours
Source: Br J Cancer. 2008 Jan 15;98(3):571–9. doi: 10.1038/sj.bjc.6604181 (PMC2243163; doi:10.1038/sj.bjc.6604181)
Supplement: Supplementary Figure 5 [file 6604181x5.doc]

**Supplemental Figure 5**

**Potential CTCF/BORIS binding sites within the promoter of the *PR* gene**

**A:** CTCF/BORIS consensus sequence as determined by Kim et al (1) is shown as DNA logo. Consensus previously reported by Chao et al (2) and Bell and Felsenfield (3) is shown in black. The “consensus” has key conserved nucleotides C, G, and G and C together

at positions 6, 11, 14, and 16; nucleotides at other positions can vary.

B. **Potential CTCF/BORIS binding sites within the promoter of the *PR* gene (-711+825).** Transcription start site B (position 0) is highlighted in yellow and transcription start site A (position + 751) is highlighted in green. Four potential CTCF binding sites are highlighted in grey.

-711

GGATCCATTT TATAAGCTCA AAGATAATTA CTTTTCAGAC TAAGAATATT TAGGGTAAAA

AGTACTGTTC AACATCTCTA CTGAGGATGT TATGATGTAG CACACTCTAT AAGCTGGAGC

TAAAGGAAAC TTTCCTTAAA GTGCTATTTA CTAAAAATTG GAACACATTC CTTAAGACAA

ATCGAAGTGT GGCACACAAC ATCCAAACTT CCATCATAGA TACAGAGGTG TTACCATCTC

CCACTCCCAA ATTTCTTTGT CACGCTGAGG ATACTCAAGA GGAGCAGGAC ATGTTGGTCG

CAGCAGGAGA AACTTGAAAG CATTCACTTT TATGGAACTC ATAAGGGAGA GAATCTCTTA

TTTAGTATCG TCCTTGATAC ATTTATTATT TTAAAAGATA ATGTAGCCAA ATGTCTTCCT

CTGTGTTAAA TCTTTACAAA ACTGAAATCT TAAAATGGTG ACAAAAATTC TACTTCTGAT

AGAATCTATT CATTTTTCCA ATTAGATAGG GCATAATTCT TAATTTGCAA AACAAAACGT

AATATGCTTA TGAGGTTCCA TCCCAAAGAA CCTGCTATTG AGAGTAGCAT TCAGAATAAC

GGGTGGAAAT GCCAACTCCA GAGTTTCAGA TCCTACCGGT AATTGGGGTA GGGAGGGGCT

TTGGGCGGGG CCTCCCTAGA GGAGGAGGCG TTGTTAGAAA GCTGTCTGGC CAGTCCACAG

CTGTCACTAA TCGGGGTAAG CCTTGTTGTA TTTGCGCGTG TGGGTGGCAT TCTCAATGAG

AACTAGCTTC ACTTGTCATT TGAGTGAAAT CTACAACCCG AGGCGGCTAG TGCTCCCGCA

CTACTGGGAT CTGAGATCTT CGGAGATGAC TGTCGCCCGC AGTACGGAGC CAGCAGAAGT

CCGACCCTTC CTGGGAATGG GCTGTACCGA GAGGTCCGAC TAGCCCCAGG GTTTTAGTGA

GGGGGCAGTG GAACTCAGCG AGGGACTGAG AGCTTCACAG CATGCACGAG TTTGATGCCA

GAGAAAAAGT CGGGAGATAA AGGAGCCGCG TGTCACTAAA TTGCCGTCGC AGCCGCAGCC

ACTCAAGTGC CGGACTTGTG AGTACTCTGC GTCTCCAGTC CTCGGACAGA AGTTGGAGAA

CTCTCTTGGA GAACTCCCCG AGTTAGGAGA CGAGATCTCC TAACAATTAC TACTTTTTCT

TGCGCTCCCC ACTTGCCGCT CGCTGGGACA AACGACAGCC ACAGTTCCCC TGACGACAGG

ATGGAGGCCA AGGGCAGGAG CTGACCAGCG CCGCCCTCCC CCGCCCCCGA CCCAGGAGGT

GGAGATCCTC CGGTCCAGCC ACATTCAACA CCCACTTTCT CCTCCCTCTG CCCCTATATT

CCCGAAACCC CCTCCTCCTT CCCTTTTCCC TCCTCCCTGG AGACGGGGGA GGAGAAAAGG

GGAGTCCAGT CGTCATGACT GAGCTGAAGG CAAAGGGTCC CCGGGCTCCC CACGTGGCGG

GCGGCCCGCC CTCCCCCGAG GTCGGATCCC CACTGC

+825

**C. The alignment of the potential four sites:** key nucleotides at positions 6, 11, 14, and 16 are boxed

**TCCACAGCTGTCAC**

**ACCCGAGGCGGCTA**

**TCCCCTGACGACAG**

**ACCAGCGCCGCCCT**

References

1. Kim TH, Abdullaev ZK, Smith AD, et al. Analysis of the vertebrate insulator protein CTCF-binding sites in the human genome. Cell 2007; 128: 1231-45.

2. Chao W, Huynh KD, Spencer RJ, Davidow LS, Lee JT. CTCF, a candidate trans-acting factor for X-inactivation choice. Science 2002; 295: 345-7.

3. Bell AC, Felsenfeld G. Methylation of a CTCF-dependent boundary controls imprinted expression of the Igf2 gene. Nature 2000; 405: 482-5.
