# Supplementary material for: BORIS, a paralogue of the transcription factor, CTCF, is aberrantly expressed in breast tumours
Source: Br J Cancer. 2008 Jan 15;98(3):571–9. doi: 10.1038/sj.bjc.6604181 (PMC2243163; doi:10.1038/sj.bjc.6604181)
Supplement: Supplementary Information [file 6604181x6.doc]

**Legends for Supplemental Figure 1**

Validation of the specificity of the chicken ant-BORIS antibody by different methods

A. *Immunohistochemistry.* The anti-BORIS antibody was pre-incubated with a 500-fold molar excess of immunizing BORIS peptides. The breast tissues sections were stained with the pre-blocked antibody and non-treated antibody followed by counter-staining with hematoxylin and eosin. Significant reduction in the staining is observed with the pre-blocked antibody. Images were taken at x40 magnification.

B. *Immunofluorescence.* The anti-BORIS antibody was pre-incubated with a 500-fold molar excess of immunizing BORIS peptides. HeLa cells were stained with the pre-blocked antibody and the non-treated antibody. Significant reduction in the staining is observed with the pre-blocked antibody.

C. *Detection by Western analysis of the recombinant BORIS proteins produced in the prokaryotic and eukaryotic systems.* The N-terminal domain of BORIS (amino acids 2-257) for expression in *E.col*i was generated by PCR using oligonucletide primers designed with restriction sites for Nde1 (5’- forward primer) and Age1 (3’- reverse primer) as follows:

5’-GGCCAAGTCCATATGGCAGCCACT-3’ (forward) and

5’-TGCAGACATCACACCGGTAGGTTCCTTT-3’ (reverse).

The restriction sites are underlined.

The pCMV6-BORIS containing the full length BORIS cDNA sequence was used as the template DNA. The PCR fragment was cloned into pDrive and the insert verified by sequencing. The DNA corresponding to the N-terminal BORIS domain was then excised from the pDrive with Nde1 and Age1 enzymes; the N- domain of BORIS was ligated into pET16b-SH3 (Cys) ~N also digested with Nde1 and Age1 (Chernukhin et al., 2000). The protein was produced in *E.coli* according to our previous report (Chernukhin et al., 2000). The identity of the protein was further confirmed by mass-spectrometry.

For the Western analysis, 20μl of cells lysates obtained from the ZR-75-1 cells transfected with 0 μg, 0.5 μg and 1 μg of pCMV6-BORIS vector expressing the full length BORIS protein, were used. We also loaded on the same gel 8.75 ng and 18.75 ng of the N-terminal domain of BORIS produced in *E.coli*. The antibody detects one major band of the endogenous BORIS and a strong band of the exogenously supplied BORIS in ZR-75-1 cells. The N-terminal domain of BORIS migrates as a 47 kDa protein, recognized by the chicken anti-BORIS antibody.

D. *Direct sequencing of the proteins in the 85 kDa area obtained after immunoprecipitation from the cell lysates with the anti-BORIS antibody.* MCF7 cells were used for immunoprecipitation with the anti-BORIS antibody; the protocol for this assay was described in our previous report (Chernukhin et al., 2000). Briefly, after incubation with the chicken anti-BORIS antibody, rabbit IgG raised against chicken IgY, were added to the lysates together with sepharose A. The immunoprecipitated proteins were resolved by SDS-PAGE, the 85 kDa band corresponding to BORIS was excised and subjected to in-gel tryptic digest. The resulting tryptic peptides were analyzed by LC-MS/MS (Bruker Esquire HTC) linked to locally installed Mascot server (http://www.matrixscience.com/). Three peptide sequences were identified which correspond to BORIS (CTCFL) - Homo sapiens (Human), Q8NI51|Uniprot.

**Reference**

Chernukhin, I.V., Shamsuddin, S., Robinson, A.F., Carne, A.F., Paul, A., El-Kady, A.I., Lobanenkov, V.V. & Klenova, E.M. (2000). Physical and functional interaction between two pluripotent proteins, the Y-box DNA/RNA-binding factor, YB-1, and the multivalent zinc finger factor, CTCF*. J Biol Ch*em, 275, 29915-21.
